# Application of urinary peptide-biomarkers in trauma patients as a predictive tool for prognostic assessment, treatment and intervention timing

**DOI:** 10.1038/s41598-024-83878-3

**Published:** 2025-01-06

**Authors:** Gökmen Aktas, Felix Keller, Justyna Siwy, Agnieszka Latosinska, Harald Mischak, Jorge Mayor, Jan Clausen, Michaela Wilhelmi, Vesta Brauckmann, Stephan Sehmisch, Tarek Omar Pacha

**Affiliations:** 1 Department of Trauma Surgery, Hannover Medical School, Carl-Neuberg St. 1, 30625 Hannover, Lower Saxony Germany; 2 Department of Internal Medicine IV (Nephrology and Hypertension), Medical University of Innsbruck, Anich St. 35, 6020 Innsbruck, Austria; 3 Mosaiques Diagnostics GmbH, Rotenburger Str 20, 30659 Hannover, Lower Saxony Germany

**Keywords:** Urine, Biomarker, Trauma, Polytrauma, Intensive care, Critical care, Proteomics, Peptides, Prediction, Diagnostic markers, Predictive markers, Trauma

## Abstract

Treatment of severely injured patients represents a major challenge, in part due to the unpredictable risk of major adverse events, including death. Preemptive personalized treatment aimed at preventing these events is a crucial objective of patient management; however, the currently available scoring systems provide only moderate guidance. Biomarkers from proteomics/peptidomics studies hold promise for improving the current situation, ultimately enabling precision medicine based on individual molecular profiles. To test the hypothesis that peptide biomarkers could predict patient outcomes in severely injured patients, we initiated a pilot study involving consecutive urine sampling (on days 0, 2, 5, 10, and 14) and subsequent peptidome analysis using capillary electrophoresis coupled to mass spectrometry (CE-MS) of 14 severely injured patients and two additional intensive care unit patients. The urine peptidomes of these patients were compared to those of age- and sex-matched controls. Moreover, previously established urinary peptide-based classifiers, CKD273, AKI204, and Cov50, were applied to the obtained peptidome data, and the association of the classifier’s scores with a combined endpoint (death and/or kidney failure and/or respiratory insufficiency) was investigated. CE-MS peptidome analysis identified 191 significantly altered peptides in severely injured patients. A consistent increase in the abundance of peptides from A1AT, AHSG, and HBA1 was observed, while peptides derived from PIGR and UROM were consistently decreased. Most of the significant peptides (adjusted p < 0.05) were from COL1A1, and most were reduced in abundance. Two of the previously defined and validated peptidomic classifiers, CKD273 and AKI204, showed significant associations with the combined endpoint, which was not observed for the routine scores generally applied in the clinics. This prospective pilot study confirmed the hypothesis that urinary peptides provide information on patient outcomes and may guide personalized interventions in severely injured patients based on individual molecular changes. The results obtained allow the planning of a well-powered prospective trial investigating the value of urinary peptides in this context in more detail.

**Supplementary Information:**

The online version contains supplementary material available at 10.1038/s41598-024-83878-3.

## Introduction

Severe trauma patients present a significant medical challenge, often requiring admission to intensive care unit (ICU) due to life-threatening nature of their injuries^1^. These patients have sustained critical injuries, often caused by incidents such as car accidents, high-energy trauma, or falls from significant heights^2^. Managing such patients mandates a multidisciplinary approach^3^. Despite intensive monitoring and interventions, their outcomes can be unpredictable due to the severity of their injuries^4^. In many cases, their condition can deteriorate significantly, become critical, and ultimately lead to death as a result of multiple organ dysfunction syndrome (MODS), a condition characterized by the failure of multiple organs that can only be managed through therapeutic interventions^5^.

When referring to polytraumatized patients, the definition by Muhr and Tscherne is frequently applied^6^. However, the term ‘polytrauma’ is used inconsistently. Therefore, a new definition of polytrauma was recently developed^7^. Today, the widely accepted definition, the New Berlin Score, characterizes polytrauma as a significant injury in at least 2 body regions with an abbreviated injury scale (AIS) score of 3 or higher and at least one pathological value for one of the following parameters: age (above 70), hypotension (systolic blood pressure < 90 mmHg), unconsciousness (Glasgow coma scale (GCS) at the scene <  = 8), acidosis (base excess <  = -6), and coagulopathy (partial thromboplastin time (PTT) >  = 40/INR >  = 1.4)^7^.

Various scoring systems, including the sequential organ failure assessment (SOFA) score, multiorgan dysfunction (MOD) score, acute physiology and chronic health evaluation II (APACHE II) score, simplified acute physiology score II (SAPS II) and therapeutic intervention scoring system (TISS) assist clinicians in evaluating the severity of the disease and determining the intensity of treatment needed^8^. However, they do not always account for trauma as the underlying cause of ICU admission^8^.

The injury severity score (ISS) and the trauma and injury severity score (TRISS) are widely used to assess a patient’s overall injury severity. Nevertheless, the variables included in these scores may not fully reflect the complexity of a patient’s condition^9^. The revised injury severity classification (RISC) score was introduced to incorporate anatomical injury descriptions and physiological indicators. It was subsequently refined into the RISC II score, which demonstrated superior performance compared to the original RISC, particularly in terms of discrimination, precision, and calibration^10^. Although the RISC II score can more efficiently used to estimate the risk of death in severely injured patients, its integration into daily intensive care practice, especially as a tool for monitoring patient progress, is challenging^11^.

The emergence of "-omics" approaches, including proteomics/peptidomics and genomics, has the potential to improve outcome prediction and optimize treatment strategies in trauma care. Changes in urine peptide patterns can provide valid insights into several organ systems^12,13^. Biomarkers such as AKI204, which was previously developed and validated in an independent cohort and shown to be useful for predicting acute kidney injury (AKI) in ICU patients^14^, and CKD273, a biomarker for detecting chronic kidney disease (CKD) that has been already applied in more than 20 studies, can predict worsening of kidney function, as shown in the prospective study^15^. Additionally, CKD273 has been associated with predicting cardiovascular outcome^16^. These biomarkers show promise for both predicting kidney damage in polytraumatized patients and for determining the time to start renal replacement therapy early^17,18^. The Cov50 classifier, developed during the COVID-19 pandemic, could predict the incidence of death and disease progression in infected patients^19^. It also demonstrated significant mortality prediction in patients without SARS-CoV-2 infection in the ICU and the general population^17–20^.

These results led to the hypothesis that urinary peptide-based biomarker classifiers may significantly enhance the ability to monitor and manage complex pathophysiological processes in polytraumatized patients. Therefore, this pilot study examined whether trauma-specific peptides can be identified in urine samples and whether urinary peptide-based classifiers may predict patient outcomes and organ failure.

## Methods

### Cohort description

This prospective, nonrandomized pilot study examined urinary peptidomic data from 16 severely injured patients recruited between January and July 2023. All adult patients aged > 18 years of both genders who had experienced primary trauma and were admitted to our Level 1 Trauma Center were included. Patients with direct traumatic consequences to the urinary tract, bladder, or kidneys were excluded due to unpredictable analysis failure. Treatment followed the current state of medical practice, in accordance with guidelines and international standards.

Each patient was informed about the study and the procedure either immediately or after regaining consciousness and capacity. In the case of a caregiver situation, the caregiver was provided with appropriate information. Informed consent was obtained retrospectively once a caregiver was available or the capacity for consent was established. If retrospective inclusion in the study was undesirable, the patient’s collected data were excluded. All participants provided written informed consent, and the study was approved by the Ethics Committee of Hannover Medical School (No: 10415_BO_S_2022).

The study was conducted in accordance with Good Clinical Practice and the Declaration of Helsinki. According to the New Berlin score, 14 of the 16 patients met the criteria for polytrauma^11^. These multiple-injury patients had an ISS ≥ 16, at least two injuries with an AIS of ≥ 3 and an indication for intensive care monitoring or surgery. The other two patients had a single injury requiring a longer hospital stay (e.g. vacuum sealing, external fixator, extensive soft tissue damage) ensuring that the entire monitoring period was completed.

### Urine sample and data collection

**Fig. 1:**
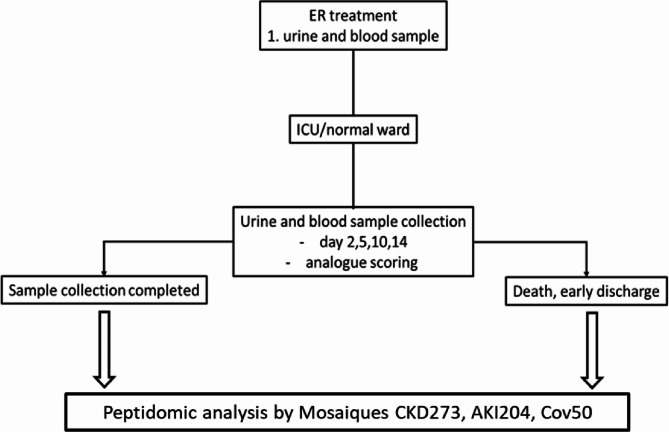
Flowchart of the study design, including urine and blood sample collection, peptidomic analysis and scoring.

For all 16 patients, urine samples and data were collected in the department of Trauma Surgery, Hannover Medical School, Hanover, Germany. The first urine sample was collected either in the emergency room, if a urinary catheter was inserted or at ICU for those patients who were directly admitted to ICU upon admission to the emergency room (Fig. 1). According to the study design, urine was sampled on days 0, 2, 5, 10, and 14 using a urine monovette containing boric acid (Sarstedt, Nümbrecht, Germany) and stored frozen at -20 °C.

### Parameters

The patients were assessed using current intensive care scores on the days of urine collection. On the day of admission (day 0), the ISS, TRISS, RISC II, APACHE-II, SAPS-II, and TIPS scores were recorded. Since the extent of injury did not change, the ISS and TRISS were only recorded at admission. APACHE-II was scored analogously on all sampling days, while SAPS-II and TIPS were assessed once upon admission.

Blood samples were also taken on the sampling days according to polytrauma standards. The samples were investigated for electrolytes, lactate, C-reactive protein (CRP), complete blood count with haemoglobin and leukocytes, coagulation parameters (INR, PTT), and kidney retention parameters.

Furthermore, the following events were recorded: long-term ventilation, tracheotomy, prone position, acute respiratory distress syndrome (ARDS), acute kidney failure, dialysis, cardiovascular events, resuscitation, death from any cause, emergency surgery, extracorporeal membrane oxygenation (ECMO), and early discharge from the ICU (Supplementary Table 1). The combined endpoint was defined as death and/or kidney failure and/or respiratory insufficiency.

### Peptidomic analysis and data processing

Urine sample preparation was performed as previously described^21^. Briefly, the urine samples were thawed immediately before use, and 0.7 ml of urine was diluted with 0.7 ml of 2 M urea and 10 mM NH4OH containing 0.02% SDS. The samples were ultrafiltered and desalted using a PD-10 column (GE Healthcare, Danderyd, Sweden) equilibrated with 0.01% NH_4_OH. The filtrate was lyophilized and stored at 4 °C until capillary electrophoresis–mass spectrometry (CE-MS) analysis was performed. CE-MS analyses were performed on a P/ACE MDQ capillary electrophoresis system (Beckman Coulter, Brea, California) coupled on-line to a micrOTOF II mass spectrometer (Bruker Daltonic, Bremen, Germany) as previously described^22^.

The mass spectral peaks were deconvoluted using MosaFinder software^23^. The obtained peak list of each polypeptide is characterized by molecular mass, CE-migration time, and normalized ion signal intensity. Normalization of signal intensities was performed using a linear regression algorithm with internal standard peptides as references. The detected peptides were deposited, matched, and annotated in the Microsoft SQL database (Microsoft, Redmond, Washington, USA).

The amino acid sequences were obtained by performing MS/MS analysis using a P/ACE CE coupled to a Q Exactive™ Plus Hybrid Quadrupole-Orbitrap™ MS instrument (Thermo Fisher Scientific, Waltham, Massachusetts, USA) as described before. The mass spectrometer was operated in data-dependent mode to automatically switch between MS and MS/MS acquisition. The data files were searched against the UniProt human nonredundant database using Proteome Discoverer 2.4 and the SEQUEST search engine without enzyme specification (activation type: HCD; precursor mass tolerance: 5 ppm; fragment mass tolerance: 0.05 Da). The high confidence peptides were defined by cross-correlation (Xcorr) > 1·9. For further validation of the obtained peptide sequences, the correlation between peptide charge at a working pH of 2 and CE migration time was used to minimize false-positive derivation rates^24^. The calculated CE migration times of the sequence candidates based on their peptide sequences (number of basic amino acids) were compared to the experimental migration times.

### Application of existing urine peptide-based biomarker classifiers

In this study, predefined urinary peptide-based classifiers were used to predict the onset and progression of CKD (CKD273), AKI (AKI204,) and critical outcome of COVID-19 (Cov50)^13,19,25^. For each classifier and patient, the numeric classification score was calculated. All three classifiers are based on support vector machines (SVM). The classifier score was calculated from the relative abundance of 273, 204 or 50 urinary peptides, as determined by a peptidomics method. Classification is performed by determining the Euclidian distance (defined as the SVM classification score) of the x-dimensional vector to the x-1-dimensional maximal margin hyperplane.

### Statistics

Statistical analysis was performed using SPSS computer software (SPSS 28, IBM, Armonk, New York, USA). Demographics, clinical variables, and peptidomics classification scores are summarized as the means ± SDs, and categorical variables are presented as frequencies (%). Continuous variables between groups were compared using the Wilcoxon rank-sum test, and categorical variables were compared using Fisher’s exact test.

The CE-MS-based data of 14 trauma patients and 14 sex- and age-matched healthy controls form the previous study extracted from the Human Urinary Proteome Database were used to identify urinary peptides potentially associated with trauma^17–20,26^. The statistical analysis was performed using R-based statistical software. Only peptides identified by their amino acid sequences were considered. P values were obtained using the Wilcoxon rank-sum test, followed by Benjamini–Hochberg false discovery rate adjustment. The correlation matric for the severity scores and urinary peptide-based classifiers was estimated using Pearson analysis. The regression analysis was performed in MedCalc (version 12.1.0.0; MedCalc Sofware, Mariakerke, Belgium) and the p-values were calculated using Steiger’s Z-test.

## Results

### Cohort characteristics

The patient characteristics of the whole cohort (n = 16) are shown in Table 1. The average age of the study population was 46 ± 21 years. The mean ISS was 27 ± 15, the average TRISS was 56 ± 39, and the average RISC II score was 11 ± 14. Table 2 shows mean AIS, systolic blood pressure and American Society of Anesthesiologists (ASA) score (classification of patients regarding their physical condition, American Society of Anesthesiologists) of the 14 polytraumatized patients. The mean AIS for the head, face, thorax, abdomen, extremities with pelvic injuries and external injuries were 2.00 ± 1.24, 0.64 ± 0.84, 2.64 ± 1.65, 2.00 ± 1.74, 2.50 ± 1.40 and 0.93 ± 0,83, respectively, at admission. The mean systolic blood pressure was 133.93 mmHg ± 17.67 at admission. The average preclinical ASA score was 1.43. Upon admission, the average SAPS II score was 32 (± 13), and the average TIPS score was 17.54 (± 9.46). The average age of the matched healthy controls was 46 ± 21 years with 8 male individuals. The healthy individuals had no impaired renal function.

**Table 1:** Baseline cohort characteristics.

Characteristic	N	Overall mean ± SD
Age (years)	16	46 ± 21
Female, n (%)		8 (50%)
Lactate (mmol/l)	14	2.76 ± 1.91
C-reactive protein (mg/dl)	16	3.1 ± 4.7
Haemoglobin (g/dl)	16	11.76 ± 2.54
CKD273	15	0.13 ± 0.64
AKI204	15	-0.14 ± 0.92
Cov50	15	-0.14 ± 1.30
APACHE 1.day	14	12 ± 8
RISC II	14	11 ± 14
ISS	16	27 ± 15
TRISS	15	56 ± 39
SAPS II	14	32 ± 13
TIPS	14	17 ± 10

Mean values for baseline cohort of all 16 patients included (14 polytrauma patients).

**Table 2:** Mean values for AIS, systolic blood pressure and ASA of all 14 polytraumatzed patients.

Characteristics	N	mean ± SD
Abbreviated Injury Scale (AIS)		
Head	14	2.00 ± 1.24
Face	14	0.64 ± 0.84
Thorax	14	2.64 ± 1.65
Abdomen	14	2.00 ± 1.74
Extremities. pelvis	14	2.50 ± 1.40
External	14	0.93 ± 0.83
Systolic blood pressure on admission (mmHg)	14	133.93 ± 17.67
ASA	14	1.43 ± 0.65

### Trauma patient outcomes

Supplementary Table 1 lists the frequencies of injuries and recorded events in the polytrauma patients (n = 14) during the 14-day monitoring period. Eleven patients suffered from traumatic brain injury (79%), while one patient had spinal cord injury (7%). Thoracic trauma was present in 71% of the patients (n = 10), and half of the patients had both abdominal trauma and pelvic injuries. The following events were observed during the study: 1 patient did not survive the study (7%). Four patients developed respiratory insufficiency (29%), and an equal number of patients suffered acute kidney failure (29%), with 1 patient requiring dialysis. ECMO support was necessary for one patient (7%). Additionally, one patient needed to be placed in the prone position during intensive care (7%). ARDS developed in 1 patient (7%). Supplementary Table 2 shows the frequencies of successful probe collection, score and blood sample trends over the entire monitoring period of 14 days. Not all urine samples could be collected during the monitoring period, mostly due to early discharge. Eight patients completed the entire study in 14 days (57%). Despite early discharge and the reduction in the number of patients from the third sampling point (Day 5), the average APACHE II score showed no consistent trend over time (12.3 ± 7.6, 11.2 ± 6.0, 10.9 ± 6.2, 12.1 ± 6.2, 12.6 ± 6.6). The highest average lactate levels were observed on the day of admission (2.8 mmol/l ± 1.9). Subsequently, there was a successive decrease, with normalization of the lactate levels on the following sampling days (1.6 mmol/l ± 1.6, 1.0 mmol/l ± 0.4, 0.8 mmol/l ± 0.3, 0.7 mmol/l ± 0.1).

On average, all included patients had normal CRP levels at admission (3.2 mg/dl ± 1.9). On sampling days 2 and 4, increases in the average values to 1695 mg/dl ± 99.46 and 139.0 mg/dl ± 166.64, respectively, were observed.

On the day of admission, the patients had an average Hb level of 11.7 g/dl ± 2.7, which showed the greatest decrease at sampling day 2 (9.1 g/dl ± 2.2) and then a successive mild decline at the remaining sampling days (9.0 g/dl ± 1.7, 8.3 g/dl ± 1.3, 8.9 g/dl ± 0.9). During the monitoring period, 3 patients did not require any packed red blood cells (PRBC) (21%), 1 patient received 4 PRBCs (7%), 4 patients each received 5 (29%), 2 patients required 7 (14%), another 2 patients received 12 (14%), and one patient required 36 while another needed 57 PRBCs (7%).

### Definition of urinary peptides specific for polytrauma patients

**Fig. 2:**
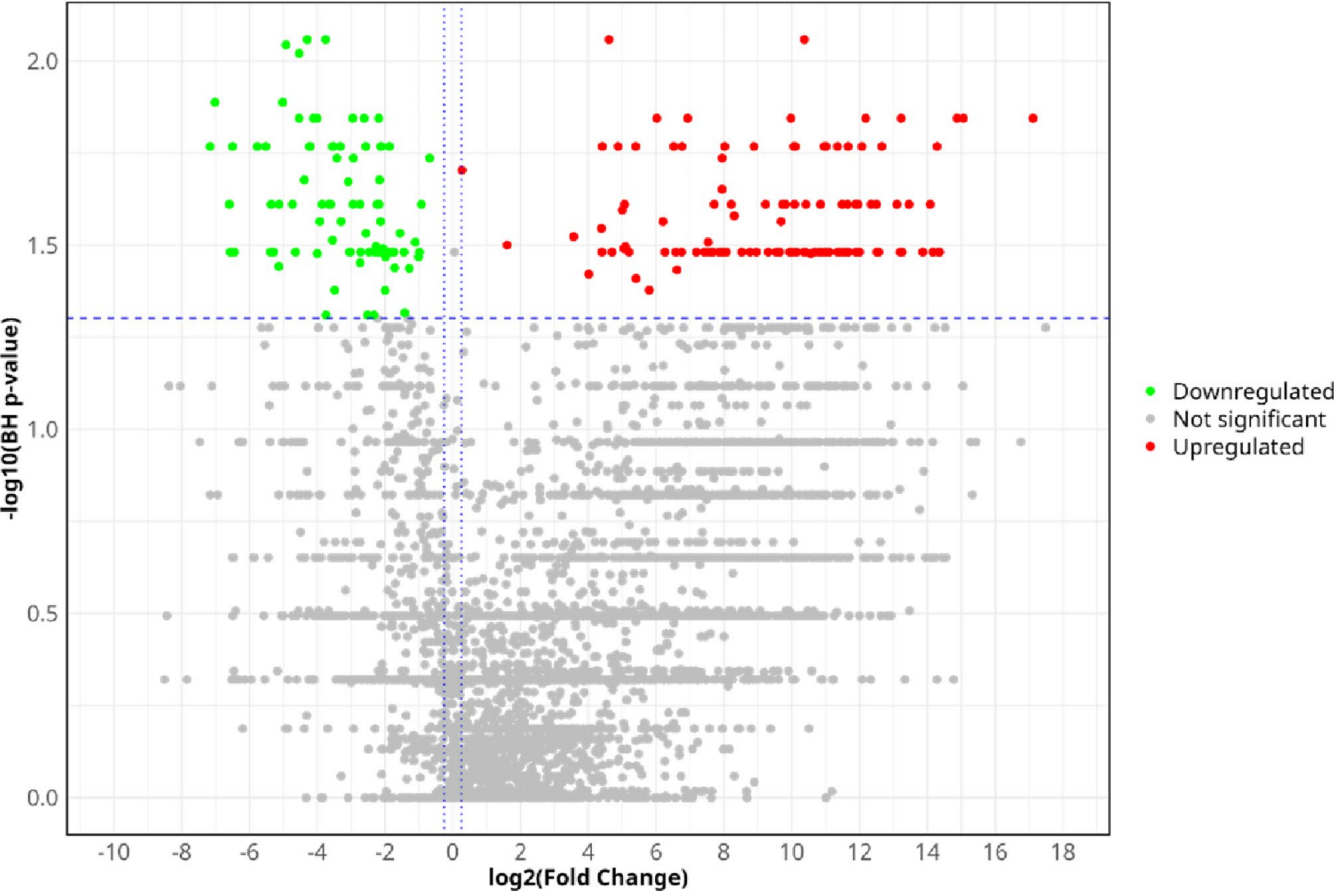
Volcano plot depicting the regulation of peptides in polytraumatized patients in relation to healthy controls. Showed are fold changes (log2) and p-values (Wilcoxon rank-sum test, followed by Benjamini–Hochberg adjustment).

A total of 19,144 different urinary peptides were detected across the peptidomic data from 16 severely injured patients. Among these, 4,573 peptides were identified by their amino acid sequences, and only these were included in the statistical analysis. The comparison of urinary peptide data from 14 polytraumatized patients with healthy controls resulted in 191 peptides significantly affected (p < 0.05, Wilcoxon test followed by Benjamini and Hochberg correction), with a fold change of at least 20%. Peptides included in the statistical analysis are shown in the form of a volcano plot in Fig. 2. All the identified peptides are listed in Supplementary Table 3. We observed a consistent increase in the abundance of the peptides from alpha-1-antitrypsin (A1AT) (n = 9), alpha-2-HS-glycoprotein (AHSG) (n = 3), and hemoglobin subunit alpha (HBA1) (n = 4), as well as decrease in the abundance of fragments from polymeric immunoglobulin receptor (PIGR, n = 5), uromodulin (UROM) (n = 4) and in 6 of 7 CD99 fragments. Most of the significant peptides were fragments of collagen alpha-1(I) (COL1A1, n = 44), 24 of which were decreased. The alignment of the increased and decreased COL1A1 fragments to the COL1A1 sequence is showed in Supplementary Fig. 1. We consistently observed an increase in the abundance of peptides from the C-termini. When comparing the 191 significantly altered peptides identified in this study with the 273, 204, and 50 peptide biomarkers included in the previously developed classifiers CKD273, AKI204, and Cov50, respectively, we found an overlap of a total of 28 peptides with 13 peptides with CKD273, 14 peptides with AKI204, and 6 peptides with Cov50 (Supplementary Table 3).

### Application of urinary peptide-based classifiers and their association with established severity scores

**Fig. 3:**
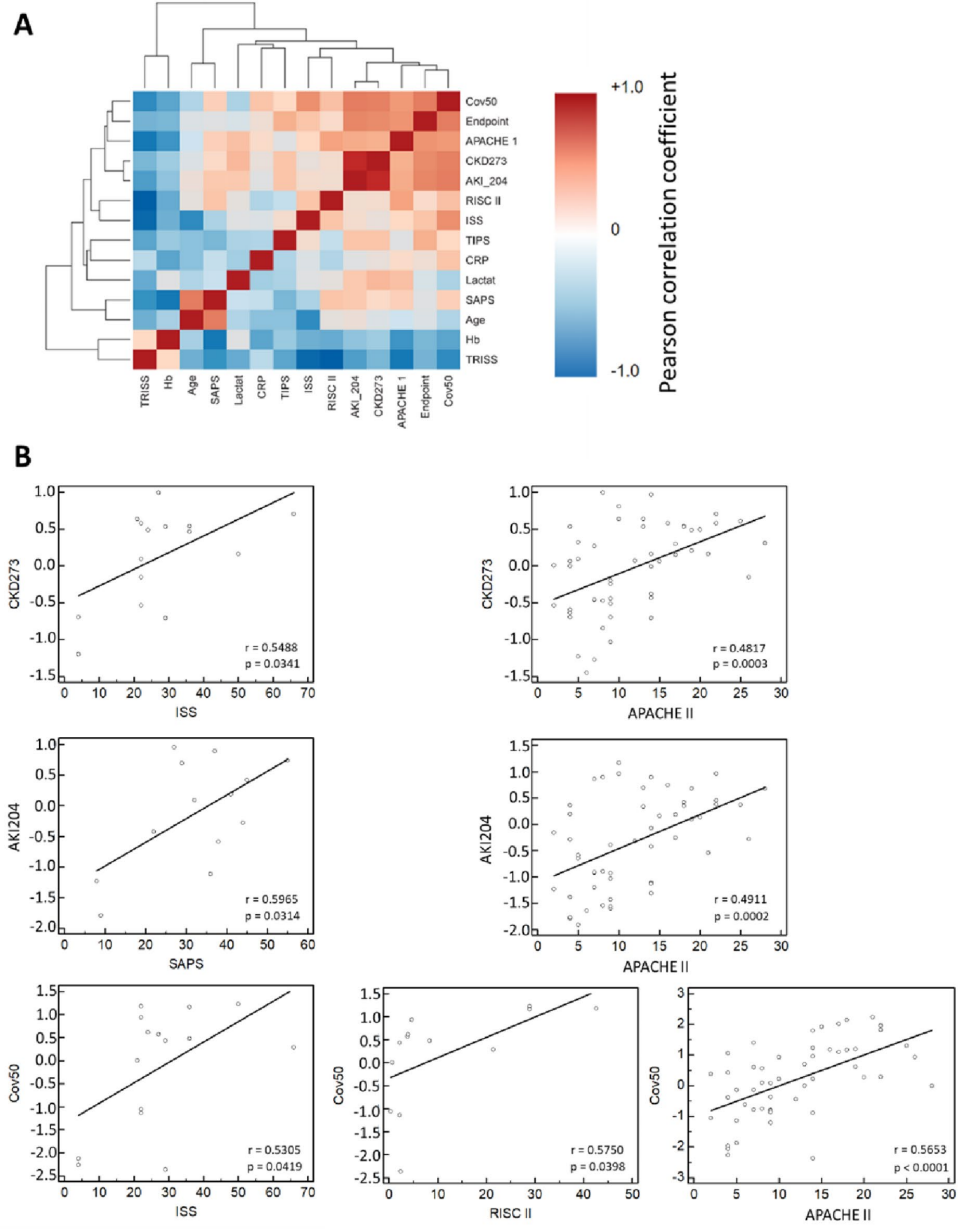
Chart of correlation statistics between the clinical parameters, ICU severity scores and peptide-based classifiers scores. (**A**) The correlation metrics based on Pearson analysis, with positive correlations shown in dark red and negative correlations in blue. (**B**) Scatter diagrams with regression lines for significant associated urinary peptide classifiers with established severity scores with the corresponding correlation coefficients (r) and p-values (Steigers Z-Test).

On the obtained CE-MS data, the previously developed classifiers CKD273, AKI204 and Cov50 were applied. The classifier for CKD prediction, CKD273, demonstrated a significant correlation (Pearson) with the ISS (r = 0.5488, p = 0.034) and the APACHE-II score (r = 0.4817, p < 0.001). The APACHE-II score was the only severity score that was obtained not only at baseline but also at every follow-up timepoint. The AKI204 classifier correlated significantly (p = 0.031) with the SAPS (r = 0.5965) and APACHE-II score (r = 0.4911, p < 0.001). The Cov50 classifier was significantly correlated with the ISS (r = 0.5305, p = 0.042), the RISC II (r = 0.5750, p = 0.040) and the APACHE-II scores (r = 0.5653, p < 0.001). Figure 3A presents a chart of correlation metrics based on Pearson analysis, with positive correlations shown in dark red and negative correlations in blue. Individual scatter plots with regression lines for urinary peptide classifiers significantly associated with established severity scores, along with correlation coefficients (r) and p-values (Steiger’s Z-test), are shown in Fig. 3B.

### Associations of the parameters with the combined endpoint

**Fig. 4:**
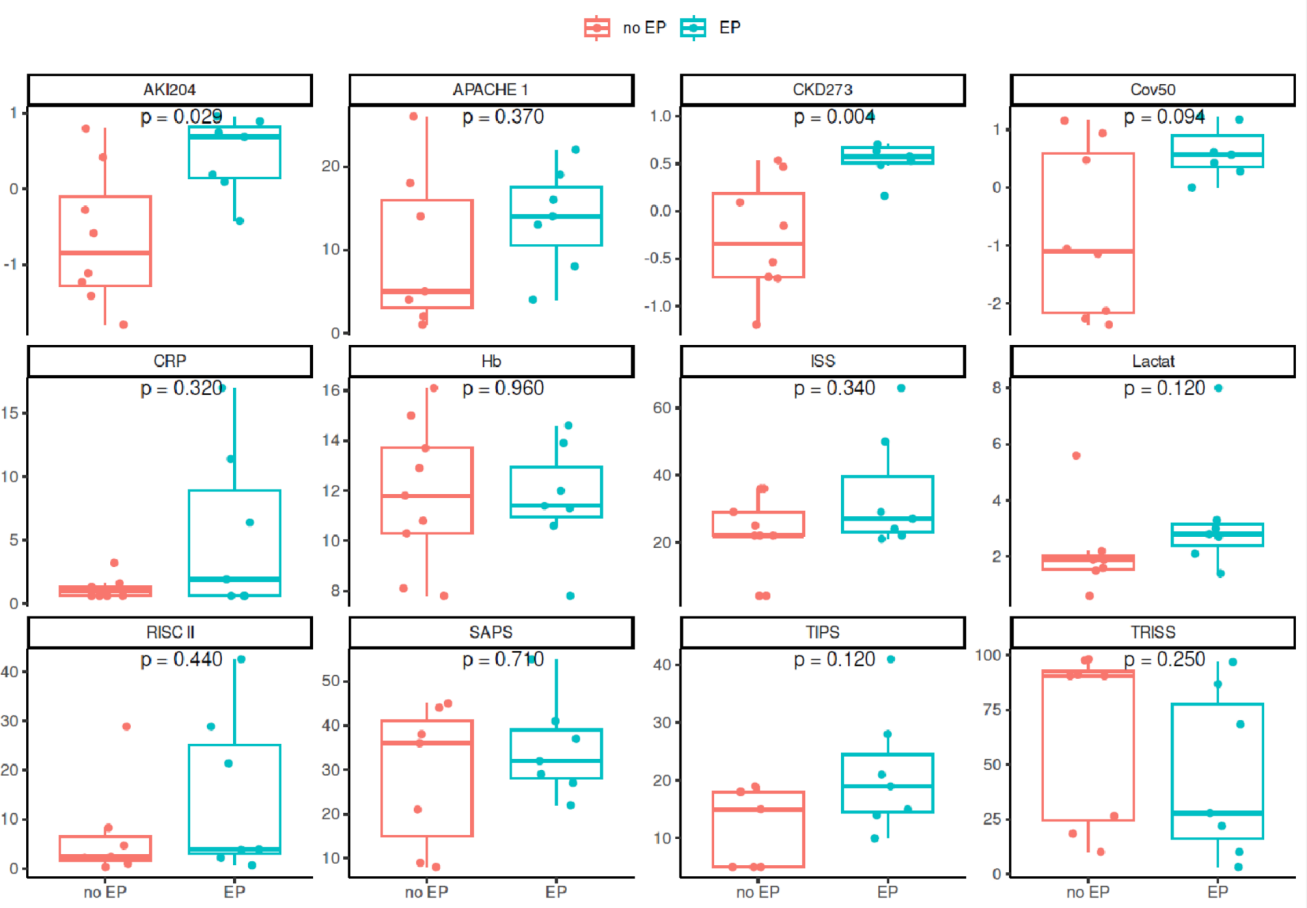
Association of the parameters with the combined endpoint. Shown are bBox-whisker plots of the baseline parameters and their associations with the prediction of the combined endpoint (EP) with the corresponding p-values (Wilcoxon rank-sum test).

**Fig. 5:**
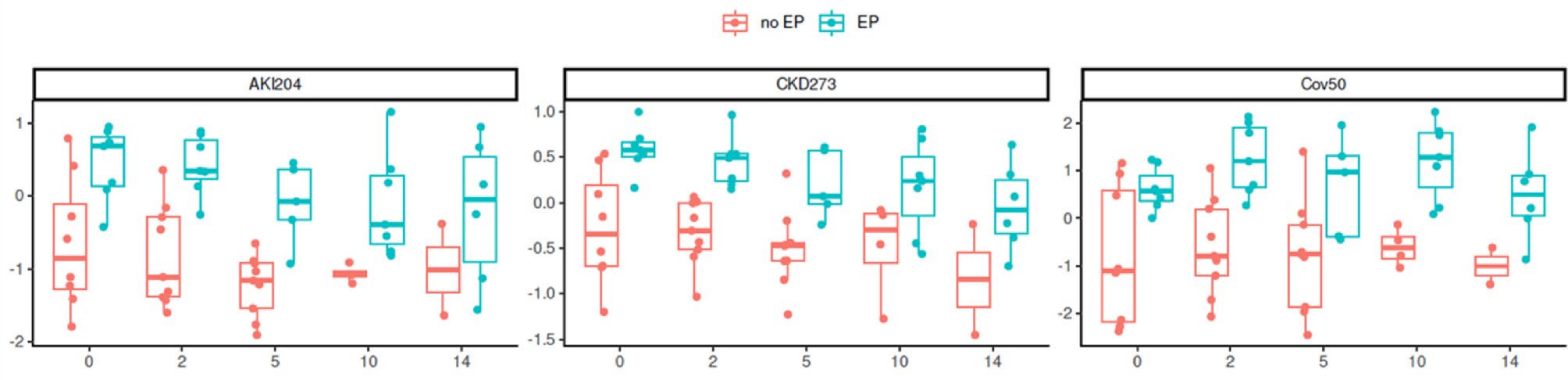
Distribution of classification scores between patients who reached the combined endpoint (EP) and those who did not throughout the entire study duration. Showed are individual classification scores (y-axis) of urinary peptide classifiers for each sampling time point (x-axis, days).

The associations between clinical parameters, severity scores, and the obtained peptide-based classification scores at baseline with the combined endpoint were investigated in the next step. The results are listed in Table 3 and presented as box-whisker plots in Fig. 4. Significant differences (Wilcoxon rank-sum test) between the patients who reached the combined endpoint and those who did not were observed for CKD273 and AKI204 (p = 0.004 and p = 0.029), while the Cov50 classifier showed only a trend (p = 0.094). The clinical parameters and severity scores did not significantly differ between the groups (p > 0.05). Figure 5 displays the classification scores of the peptide-based classifiers throughout the entire study period.

**Table 3:** Parameter associations with the combined endpoint.

Characteristic	no EP, N = 9	EP, N = 7	p value*
Age (years), mean ± SD	43 ± 15	49 ± 28	0.8
Female gender, n (%)	5 ± 56%	3 ± 43%	> 0.9
Lactate (mmol/l), mean ± SD	2.19 ± 1.59	3.33 ± 2.15	0.12
C-reactive protein (mg/dl), mean ± SD	1.2 ± 0.8	5.5 ± 6.5	0.3
Haemoglobin (g/dl), mean ± SD	11.83 ± 2.88	11.66 ± 2.24	> 0.9
CKD273, mean ± SD	-0.27 ± 0.61	0.58 ± 0.25	**0.004**
AKI204, mean ± SD	-0.65 ± 0.91	0.45 ± 0.51	**0.029**
Cov50, mean ± SD	-0.80 ± 1.46	0.61 ± 0.45	0.094
APACHE 1.day, mean ± SD	10 ± 10	14 ± 6	0.4
RISC II, mean ± SD	7 ± 10	15 ± 16	0.4
ISS, mean ± SD	22 ± 12	34 ± 17	0.3
TRISS, mean ± SD	65 ± 39	45 ± 38	0.2
SAPS II, mean ± SD	29 ± 16	35 ± 11	0.7
TIPS, mean ± SD	12 ± 7	21 ± 10	0.13

EP =Endpoint, *Wilcoxon rank-sum test (for continuous variables); Fisher’s exact test (for categorical variables).

Significant are in value [bold].

## Discussion

The aim of this pilot study was to investigate whether severely injured trauma patients exhibit significant alterations in urinary peptides. Furthermore, the study examined whether the classifiers based on the urinary peptide signatures could serve as predictive biomarkers for patient outcomes, potentially offering valuable insights to support personalized treatment strategies and guide clinical decision-making.

Our findings revealed significant alterations in urinary peptide levels among trauma patients, with 191 peptides showing significant changes. While some of these peptides were derived from blood proteins, potentially indicating albuminuria, this does not fully explain the findings. Many peptides, particularly those derived from COL1A1, showed decreased abundance, in contrast to typical patterns observed in albuminuria or proteinuria, where increased levels of affected proteins and peptides are more common.

A substantial proportion of the altered peptides originated from collagen or kidney-specific proteins, suggesting that mechanism beyond albuminuria contribute to these changes. In fact, the most notable changes were observed in collagen peptides. This was expected, as collagen peptides are the most abundant peptides in urine and are influenced by several pathologies, including kidney and cardiovascular diseases, as well as increased mortality risk^24^. However, the changes in collagen were not uniform: some collagen peptides increased significantly, while others decreased. The reasons for these mixed effects remain mainly unclear. However, recent findings suggest the possibility of sequential peptide degradation^27^, beginning with the initial cleavage of collagen by endopeptidases, followed by further breakdown of peptides through nonspecific exopeptidases. This secondary degradation may introduce greater variability, potentially explaining the observed inconsistencies in collagen peptide regulation.

Furthermore, significant changes were observed in peptides derived from A1AT, AHSG and HBA1. Similar alterations have been reported in patients with chronic or acute kidney diseases, suggesting these changes may reflect severe renal stress in trauma patients^16,22,28^. Additional evidence of kidney damage is provided by the consistent reduction of uromodulin fragments^29^. This finding is in line with our expectations, as severely injured patients often suffer from kidney damage, which can progress to acute renal failure requiring dialysis^30^. Other changes included a consistent reduction in fragments of CD99 and the PIGR^31^. Similar alterations have been observed in severe of fatal cases of COVID-19, where they were interpreted as potential markers of endothelial damage^32,33^. Additionally, reduced PIGR expression has been reported in the lung tissue of patients with chronic obstructive pulmonary disease, where it was suggested to be linked to TGF-β –driven reprogramming of airway epithelial cells^34^. Moreover, findings from the FROG-ICU study revealed urinary peptidomic signatures in critically ill patients that were similar between those with subclinical AKI and overt AKI^35^, and reflected inflammation, hemolysis, and endothelial dysfunction, and were associated with 1-year mortality. These similarities further underscore the potential of urinary peptide analysis in identifying systemic complications and predicting outcomes in severely ill or injured patients.

There is a substantial need for such predictive tools in the ICU, as current injury or mortality scores (ISS, TRISS, RISC II) and progression assessments like the APACHE II score do not adequately predict or guide interventions such as dialysis. The outcomes rely on the rapid detection of the condition and the continuous, rigorous monitoring of patients^36^. Numerous efforts are underway to predict AKI in both medically ill and severely injured patients^37^. For example, many functional tests aim to make predictions not through the quantification of laboratory chemical parameters, but by assessing urine output after stress tests, such as the furosemide stress test or renal functional reserve^37^. Still, such testing requires a coordinated timing of furosemide administration and documentation of time and urine output^38^, which must be integrated into the time-intensive care of critically ill patients (40).

Our study showed significant changes in CKD273 and AKI204 scores, which were measured noninvasively based on changes in urinary peptides. These changes were observed in polytrauma patients with combined endpoint of death and/or kidney failure and/or respiratory insufficiency.

Since urinary peptidomics involves a comprehensive, untargeted analysis of endogenous peptides, it is particularly valuable for clinical applications, as diseases often exhibit high molecular heterogeneity and complex underlying pathophysiological processes. This pilot study demonstrated a significant prediction probability for the development of AKI in polytraumatized patients using urinary peptide classifiers. Moreover, CKD273 showed a significant correlation with the ISS, reflecting the severity of patients’ injuries. Likewise, it was correlated with the APACHE II-Score, which was the only score collected at all sampling points and provides insights into disease progression. Similarly, AKI204 showed a significant correlation with SAPS II and the APACHE II scores, and could provide an outlook on the intensive care course and care requirements.

In the context of ARDS the Berlin Definition is currently used as the gold standard for ARDS treatment^39^, relying on various criteria that must be fulfilled to make a diagnosis^39^. However, clinicians are often confronted with the clinical picture of ARDS unexpectedly. The silent period before onset can lead to irreversible complications, and delayed intervention^39^, indicating a need for biomarkers to detect ARDS early and guide treatment. Although the courses of Covid-19 and classical ARDS differ in some pathophysiological aspects, they do have similarities. Especially through the development of ARDS in severe Covid cases, these adverse outcomes can be identified by the urinary peptidomic Cov50 marker. In our study, out of 10 (71.4%) patients with thoracic trauma, 4 developed respiratory insufficiency (28.6%), of which only one patient manifested ARDS (7.1%). The Cov50 showed only a trend in predicting the combined endpoints but did not reach statistical significance. Thus, to better evaluate the suitability of urinary peptidome, a larger patient cohort is needed, compared to this pilot study.

Due to the small number of patients and low incidence of ARDS at the time of the pilot study, no reliable conclusions could be drawn regarding prognostic assessment, treatment interventions, and intervention timing, such as determining when ECMO (7.1%) or prone positioning (7.1%) should be initiated. Nevertheless, considering the trend of Cov50 alongside other peptidomic results, it appears to be a promising avenue to address these issues in the management of severe ARDS cases. Similar to CKD273, Cov50 also showed a significant correlation with the ISS, as well as with the RISC II and the APACHE II-Score. Thus, Cov50 could also serve as a classifier for the primary assessment of injury severity and the resulting mortality risk.

Within our study several scores were estimated for the severity and to predict survival probability in polytraumatized patients. However, none of the established scores showed a significant association with the combined endpoint, likely due to the limited power of the study. Nevertheless, these clinical severity scores are a gold standard in their respective areas of use and are employed daily in the assessment and care of severely injured and critically ill intensive care patients.

This study has several limitations. First, the sample size was small, with a total of 16 patients, of whom 14 had polytrauma. As a result, this study was designed as a pilot investigation. Additionally, the complete set of samples (14 days, 5 samples per patient) could only be collected from 8 patients. Furthermore, due to the small number of patients, the study exhibited highly variable pattern of injuries and injury severity, and not all relevant laboratory values could be collected on all sampling days. Additionally, data collection became more challenging once patients were transferred from the ICU to the general ward.

## Conclusion

The prediction of severe organ damage/organ failure and their critical courses in severely injured patients is still associated with high uncertainty due to individual patterns of injury and progression. The prediction of these events is frequently based on the expertise and experience of the intensivist, with frequent laboratory tests and close monitoring of important organ systems, such as the liver, kidneys, heart, and lungs, required to prepare for unexpected courses and indications for interventions. Nonetheless, intensive care physicians are often faced with unforeseen developments.

This pilot study aims to serve as a proof of concept for applying peptidome analysis in traumatology, potentially paving the way for a multicenter study, as a significantly larger number of cases is required for a conclusive evaluation of the investigated biomarkers.

## Electronic supplementary material

Below is the link to the electronic supplementary material.

Supplementary Material 1

## Data Availability

The datasets generated and analysed during the current study are not publicly available but are available from the corresponding author upon reasonable request.

## Abbreviations

AHSG: Alpha-2-HS-glycoprotein
AIS: Abbreviated injury scale
A1AT: Alpha-1-antitrypsin
AKI: Acute kidney injury
APACHE II: Acute physiology and chronic health evaluation II
ARDS: Acute respiratory distress syndrome
ASA: American Society of Anesthesiologists
CE-MS: Capillary electrophoresis–mass spectrometry
CKD: Chronic kidney disease
COL1A1: Collagen alpha-1(I)
CRP: C-Reaktive protein
ECMO: Extracorporeal membrane oxygenation
GCS: Glasgow coma scale
Hb: Haemoglobin
HBA1: Hemoglobin subunit alpha
ICU: Intensive care unit
ISS: Injury severity score
MODS: Multiple organ dysfunction syndrome
MOD: Multiorgan dysfunction
SOFA: Sequential organ failure assessment
PRBCs: Packed red blood cells
PTT: Partial thromboplastin time
PIGR: Polymeric immunoglobulin receptor
RISC: Revised injury severity classification
RTS: Revised trauma score
SAPS II: Simplified acute physiology score
TISS: Therapeutic intervention scoring system
TRISS: Trauma and injury severity score
UROM: Uromodulin
