# Supplementary material for: Application of urinary peptide-biomarkers in trauma patients as a predictive tool for prognostic assessment, treatment and intervention timing
Source: Sci Rep. 2025 Jan 6;15:898. doi: 10.1038/s41598-024-83878-3 (PMC11704255; doi:10.1038/s41598-024-83878-3)

Supplementary data

Supplementary table 1: Frequencies of injuries and events in the N=14 patients within the trauma cohort during the 14-day monitoring period

Characteristics	Frequency (N)	Percentage
Traumatic brain injurie	11	78.6
Spinal cord injurie	1	7.1
Thoracic injurie	10	71.4
Abdominal injurie	7	50
Pelvic injurie	7	50
Respiratory insufficiency	4	28.6
ARDS	1	7.1
Prone position	1	7.1
ECMO	1	7.1
Akute kidney injurie	4	28.6
Dialysis	1	7.1
Cardiovascular Event	0	0
Resuscitation	0	0
Death	1	7.1
Monitoring of 14 days completed	8	57.1
Early discharge	6	42.9

Supplementary table 2: Frequencies of successful sample collection, score and blood sample trends during the monitoring period of 14 days

Characteristics	Total	Frequency	Percentage
1. Sample collection on admission	14	14	100
2. Sample collection on day 2	14	14	100
3. Sample collection on day 5	14	12	85.7
4. Sample collection on day 10	14	9	64.3
5. Sample collection on day 14	14	8	57.1
**Characteristics**	**Total**	**Mean**	**SD**
1. APACHE II score on admission	14	12.29	7.64
2. APACHE II score on day 2	14	11.21	6.02
3. APACHE II score on day 5	12	10.92	6.24
4. APACHE II score on day 10	9	12.11	6.21
5. APACHE II score on day 14	8	12.57	7.66
1. Lactate on admission	14	2.76	1.91
2. Lactate on day 2	13	1.59	1.59
3. Lactate on day 5	10	0.96	0.35
4. Lactate on day 10	5	0.82	0.27
5. Lactate on day 14	3	0.73	0.12
1. CRP on admission	14	3.20	5.02
2. CRP on day 2	14	169.48	99.46
3. CRP on day 5	13	104.01	90.20
4. CRP on day 10	9	138.98	166.64
5. CRP on day 14	7	56.39	38.97
1. Hb on admission	14	11.69	2.66
2. Hb on day 2	14	9.06	2.18
3. Hb on day 5	13	9.02	1.71
4. Hb on day 10	9	8.32	1.32
5. Hb on day 14	7	8.89	0.88

Supplementary table 3: List of urinary peptides specific for polytrauma patients.

unaj,wilcox-p-value	AUC	p-value BH	Frequency Trauma	mean Trauma	Frequency controls	mean controls	Fold change	Increased abundance in cases=1	Sequence	ProteinName	Start AA	Stop AA	Uniprot Accession	Symbol	Xcorr	CKD273 (1=peptide included in the classifier)	AKI204 (1=peptide included in the classifier)	Cov50 (1=peptide included in the classifier)
1.22E-03	0.83	3.30E-02	0.71	788.38	0.14	29.43	26.79	1	VGMGQKDSYVGDEAQSKRGILT	Actin. cytoplasmic 2	45	66	P63261	ACTG1	5.01			
4.07E-04	0.87	2.45E-02	0.21	2.36	0.79	88.17	0.03	0	EKTPVSDR	Albumin	489	496	P02768	ALB	2.31			
1.39E-03	0.79	3.30E-02	0.57	21052.79	0.00	0.00	undefined	1	LVELVKHKPKATKEQL	Albumin	553	568	P02768	ALB	2.40			
5.26E-04	0.84	2.45E-02	0.71	41523.70	0.07	2.68	15493.92	1	MIEQNTKSPLFMGKVVNPTQK	Alpha-1-antitrypsin	398	418	P01009	SERPINA1	7.82	1	1	
1.39E-03	0.79	3.30E-02	0.57	2013.47	0.00	0.00	undefined	1	VKELDRDT	Alpha-1-antitrypsin	197	204	P01009	SERPINA1	2.29			
1.39E-03	0.79	3.30E-02	0.57	802.73	0.00	0.00	undefined	1	SLGTKADTHDEIL	Alpha-1-antitrypsin	89	101	P01009	SERPINA1	3.00			
1.39E-03	0.79	3.30E-02	0.57	6073.80	0.00	0.00	undefined	1	EAIPMSIPPEVKFNKPFV	Alpha-1-antitrypsin	378	395	P01009	SERPINA1	4.13		1	
6.65E-05	0.89	1.43E-02	0.79	34392.86	0.00	0.00	undefined	1	EAIPMSIPPEVKFNKPFVF	Alpha-1-antitrypsin	378	396	P01009	SERPINA1	4.13			
1.95E-04	0.86	1.71E-02	0.71	20007.54	0.00	0.00	undefined	1	LMIEQNTKSPLFMGKVVNPTQK	Alpha-1-antitrypsin	397	418	P01009	SERPINA1	8.00		1	
1.39E-03	0.79	3.30E-02	0.57	436.22	0.00	0.00	undefined	1	IFFKGKWERPFEVKDTEEEDF	Alpha-1-antitrypsin	212	232	P01009	SERPINA1	7.55		1	
1.39E-03	0.79	3.30E-02	0.57	20644.70	0.00	0.00	undefined	1	FLPDEGKLQHLENELTHDIITKF	Alpha-1-antitrypsin	277	299	P01009	SERPINA1	6.10		1	

1.39E-03	0.79	3.30E-02	0.57	3857.72	0.00	0.00	undefined	1	FGDTEEAKKQINDYVEKGTQGKIVDLVKELDRDT	Alpha-1-antitrypsin	171	204	P01009	SERPINA1	12.58		1	
9.10E-04	0.85	3.30E-02	0.86	24927.30	0.21	230.64	108.08	1	VVSLGSPSGEVSHPRKT	Alpha-2-HS-glycoprotein	323	339	P02765	AHSG	6.22	1	1	
5.83E-06	1.00	8.74E-03	1.00	20349.77	0.43	14.33	1420.08	1	MGVVSLGSPSGEVSHPRKT	Alpha-2-HS-glycoprotein	321	339	P02765	AHSG	5.94	1	1	
1.39E-03	0.79	3.30E-02	0.57	268.31	0.00	0.00	undefined	1	MGVVSLGSPSGEVSHPR	Alpha-2-HS-glycoprotein	321	337	P02765	AHSG	4.85			
5.61E-04	0.88	2.54E-02	0.86	5178.29	0.64	160.79	32.21	1	AILDETKGDYEK	Annexin A1	326	337	P04083	ANXA1	3.06			
1.39E-03	0.79	3.30E-02	0.57	1362.82	0.00	0.00	undefined	1	RQKLGPHAGDVEGHLSFLEKDLRDK	Apolipoprotein A-IV	326	350	P06727	APOA4	8.67			
1.95E-04	0.86	1.71E-02	0.71	259.18	0.00	0.00	undefined	1	mENGKIKVLNQEL	Apolipoprotein D	69	81	P05090	APOD	3.68			
1.88E-03	0.80	4.19E-02	0.07	0.63	0.64	17.29	0.04	0	KVEHSDLS	Beta-2-microglobulin	68	75	P61769	B2M	2.25			
1.39E-03	0.79	3.30E-02	0.57	1840.29	0.00	0.00	undefined	1	VSGFHPSDIEVD	Beta-2-microglobulin	47	58	P61769	B2M	3.24			
5.36E-04	0.82	2.45E-02	0.64	296.68	0.00	0.00	undefined	1	NGERIEKVEHSDLS	Beta-2-microglobulin	62	75	P61769	B2M	3.92			
1.39E-03	0.79	3.30E-02	0.57	18401.27	0.00	0.00	undefined	1	LLKNGERIEKVEHSDLSFSKDWS	Beta-2-microglobulin	59	81	P61769	B2M	8.19	1	1	
2.27E-05	0.95	1.29E-02	0.14	23.98	0.93	806.83	0.03	0	DGGFDLSDALPDNENKKPtAIP	CD99 antigen	23	44	P14209	CD99	2.51			
6.38E-04	0.84	2.73E-02	0.07	2.79	0.71	56.43	0.05	0	ADGVSGGEGKGGSDGGGSHRKEGEEADAPGVIPG	CD99 antigen	96	129	P14209	CD99	5.89			
3.02E-04	0.89	2.13E-02	0.29	20.55	0.86	181.67	0.11	0	DGVSGGEGKGGSDGGGSHRKEGEE	CD99 antigen	97	120	P14209	CD99	5.73			
2.45E-04	0.90	1.83E-02	0.36	112.23	0.93	863.15	0.13	0	DLADGVSGGEGKGGSDGGGSHRKEGEEADAPGVIPGIVGAVV	CD99 antigen	94	135	P14209	CD99	6.34		1	

4.20E-04	0.89	2.45E-02	0.43	228.95	0.93	1531.65	0.15	0	DGVSGGEGKGGSDGGGSHRKEGEEADAPGVIPG	CD99 antigen	97	129	P14209	CD99	7.60	1		
5.09E-05	0.95	1.43E-02	0.50	205.67	1.00	937.35	0.22	0	DGVSGGEGKGGSDGGGSHRKEGEEADAPGVIPGIVGAVV	CD99 antigen	97	135	P14209	CD99	5.64		1	
1.39E-03	0.79	3.30E-02	0.57	203.66	0.00	0.00	undefined	1	DDPRPPNPPKPMPNP	CD99 antigen	65	79	P14209	CD99	2.25			
1.39E-03	0.79	3.30E-02	0.00	0.00	0.57	23.92	0.00	0	DGEAGKpGRp	Collagen alpha-1(I) chain	232	241	P02452	COL1A1	2.62			
1.56E-03	0.80	3.61E-02	0.07	0.59	0.64	54.81	0.01	0	DDGEAGKPG	Collagen alpha-1(I) chain	231	239	P02452	COL1A1	2.27			
1.34E-04	0.89	1.71E-02	0.07	3.47	0.79	242.47	0.01	0	DGQpGAKGEpGDAGAKG	Collagen alpha-1(I) chain	820	836	P02452	COL1A1	5.26	1		
3.80E-05	0.92	1.43E-02	0.07	0.69	0.86	38.13	0.02	0	DKGESGPSGP	Collagen alpha-1(I) chain	780	789	P02452	COL1A1	2.59			
2.94E-04	0.87	2.10E-02	0.14	5.35	0.79	131.34	0.04	0	DGQPGAKGEpGDAGAKGD	Collagen alpha-1(I) chain	820	837	P02452	COL1A1	4.54			
1.41E-03	0.83	3.33E-02	0.21	9.61	0.79	168.71	0.06	0	GPDGKTGpPGPA	Collagen alpha-1(I) chain	548	559	P02452	COL1A1	4.33	1		
5.26E-05	0.94	1.43E-02	0.36	39.22	0.93	644.31	0.06	0	GADGQpGAKGEpGDAGAKGDAGPpGP	Collagen alpha-1(I) chain	818	843	P02452	COL1A1	9.51			
7.65E-06	0.98	8.74E-03	0.14	41.64	1.00	573.62	0.07	0	PpGEAGKpGEQGVP	Collagen alpha-1(I) chain	651	664	P02452	COL1A1	4.11			
1.16E-04	0.90	1.71E-02	0.14	19.29	0.86	236.94	0.08	0	ESGREGApGAEGSpGRD	Collagen alpha-1(I) chain	1011	1027	P02452	COL1A1	3.85			
2.12E-04	0.89	1.71E-02	0.21	21.52	0.86	260.75	0.08	0	DGQPGAKGEpGDAGAKG	Collagen alpha-1(I) chain	820	836	P02452	COL1A1	5.10			
2.39E-04	0.90	1.83E-02	0.36	36.44	0.86	399.63	0.09	0	DGQpGAKGEpGDAG	Collagen alpha-1(I) chain	820	833	P02452	COL1A1	3.50			
1.24E-03	0.85	3.30E-02	0.29	12.47	0.86	110.31	0.11	0	DGAKGDAGApGApG	Collagen alpha-1(I) chain	706	719	P02452	COL1A1	3.88			

1.24E-03	0.85	3.30E-02	0.29	58.61	0.86	483.20	0.12	0	DDGEAGKpG	Collagen alpha-1(I) chain	231	239	P02452	COL1A1	2.47		1	
2.24E-03	0.83	4.90E-02	0.36	43.53	0.86	253.60	0.17	0	GPpGSpGEQGPSGASGP	Collagen alpha-1(I) chain	1121	1137	P02452	COL1A1	2.94			
1.14E-03	0.86	3.30E-02	0.50	73.23	0.93	409.47	0.18	0	SpGENGApGQmGPRG	Collagen alpha-1(I) chain	291	305	P02452	COL1A1	3.81			
2.24E-03	0.83	4.90E-02	0.36	61.67	0.93	313.94	0.20	0	GADGQPGAKGEpGDAGAKGDAGPpGPAGP	Collagen alpha-1(I) chain	818	846	P02452	COL1A1	7.82			
9.31E-04	0.87	3.30E-02	0.57	234.03	0.93	1152.02	0.20	0	ApGDRGEPGPpGP	Collagen alpha-1(I) chain	798	810	P02452	COL1A1	4.32			
9.88E-04	0.86	3.30E-02	0.43	209.92	0.93	957.89	0.22	0	DGSpGAKGDRGETGPA	Collagen alpha-1(I) chain	1027	1042	P02452	COL1A1	4.27			
2.02E-04	0.91	1.71E-02	0.57	252.27	1.00	1101.37	0.23	0	GAPGpQGFQGppGEPGEPGASGPMGPRGPPG	Collagen alpha-1(I) chain	194	224	P02452	COL1A1	2.87			
1.14E-03	0.86	3.30E-02	0.50	221.82	0.93	893.20	0.25	0	ADGQPGAKGEpGDAGAKGDAGPPGP	Collagen alpha-1(I) chain	819	843	P02452	COL1A1	9.01			
1.58E-03	0.84	3.64E-02	0.21	88.83	0.93	293.75	0.30	0	ADGQpGAKGEpGDAGAKGDAGPpGpA	Collagen alpha-1(I) chain	819	844	P02452	COL1A1	5.72	1		
9.63E-04	0.87	3.30E-02	0.57	487.00	1.00	1325.23	0.37	0	GANGApGNDGAKGDAGApGApGSQGApG	Collagen alpha-1(I) chain	698	725	P02452	COL1A1	4.64			
2.18E-03	0.84	4.83E-02	0.64	2500.17	1.00	6686.01	0.37	0	NGApGNDGAKGDAGApGApGSQGApG	Collagen alpha-1(I) chain	700	725	P02452	COL1A1	5.49			
2.42E-04	0.91	1.83E-02	0.57	516.21	1.00	829.32	0.62	0	PpGEAGKpGEQGVp	Collagen alpha-1(I) chain	651	664	P02452	COL1A1	3.88			
7.94E-04	0.86	3.16E-02	1.00	12755.92	1.00	4201.34	3.04	1	ApGDRGEpGPpGPAG	Collagen alpha-1(I) chain	798	812	P02452	COL1A1	4.20			
7.27E-04	0.88	3.00E-02	1.00	4968.46	0.79	419.28	11.85	1	GpPGpPGPpGPpS	Collagen alpha-1(I) chain	1181	1193	P02452	COL1A1	5.19			
1.24E-04	0.93	1.71E-02	0.93	21761.13	0.86	1020.48	21.32	1	GPpGpPGpPGPPGPPS	Collagen alpha-1(I) chain	1178	1193	P02452	COL1A1	5.42			

4.84E-06	0.95	8.74E-03	1.00	53681.52	1.00	2199.41	24.41	1	pPGpPGPpGpPGPPS	Collagen alpha-1(I) chain	1179	1193	P02452	COL1A1	4.96			
2.13E-04	0.89	1.71E-02	1.00	17445.38	0.93	592.32	29.45	1	AGPpGAPGApGAPGpVGPAGKSGDRGETGP	Collagen alpha-1(I) chain	1042	1071	P02452	COL1A1	9.63	1		
8.16E-04	0.87	3.19E-02	0.86	20320.67	0.71	593.83	34.22	1	ERGSpGPAGPKGSpGEAGRpGEAGLpGAKG	Collagen alpha-1(I) chain	510	539	P02452	COL1A1	7.27	1		
1.73E-03	0.84	3.90E-02	0.93	156828.43	1.00	3697.73	42.41	1	GPpGpPGPpGpPGPPS	Collagen alpha-1(I) chain	1178	1193	P02452	COL1A1	5.91			
1.64E-04	0.88	1.71E-02	0.79	379.88	0.07	8.04	47.25	1	GPpGpPGPAGKEG	Collagen alpha-1(I) chain	893	905	P02452	COL1A1	3.53			
1.87E-03	0.85	4.19E-02	0.86	8974.01	0.79	160.60	55.88	1	AGpPGApGAPGpVGPAGKSGDRGETGP	Collagen alpha-1(I) chain	1042	1071	P02452	COL1A1	6.91	1		
9.87E-04	0.87	3.30E-02	0.93	22601.62	0.71	234.80	96.26	1	GPpGPpGPpGPpGPPSA	Collagen alpha-1(I) chain	1178	1194	P02452	COL1A1	5.11			
6.38E-04	0.84	2.73E-02	0.71	258.05	0.07	2.52	102.40	1	ARGNDGATGAAGPpGPTGPAGPPGFPGAVGAKGEAGpQGPRGSEGPQG	Collagen alpha-1(I) chain	321	368	P02452	COL1A1	5.96			
9.70E-05	0.91	1.71E-02	0.86	1675.07	0.21	14.44	116.00	1	ATGFpGAAGRVGPpGPSGNAGPpGPpGPAG	Collagen alpha-1(I) chain	873	902	P02452	COL1A1	4.88			
1.95E-04	0.86	1.71E-02	0.71	1068.24	0.00	0.00	undefined	1	EpGKAGERGVPGPPGA	Collagen alpha-1(I) chain	591	606	P02452	COL1A1	3.10			
6.65E-05	0.89	1.43E-02	0.79	30164.66	0.00	0.00	undefined	1	GPpGPpGpPGPpGPPSAG	Collagen alpha-1(I) chain	1178	1195	P02452	COL1A1	5.17			
1.39E-03	0.79	3.30E-02	0.57	5826.56	0.00	0.00	undefined	1	GEPGKQGPSGASGERGPpGP	Collagen alpha-1(I) chain	980	999	P02452	COL1A1	5.57			
1.39E-03	0.79	3.30E-02	0.57	188.88	0.00	0.00	undefined	1	GPSGEpGKQGpSGASGERGpPG	Collagen alpha-1(I) chain	977	998	P02452	COL1A1	2.90			
1.95E-04	0.86	1.71E-02	0.71	6461.51	0.00	0.00	undefined	1	ERGSPGPAGPKGSpGEAGRpGEAGLpGAKG	Collagen alpha-1(I) chain	510	539	P02452	COL1A1	3.90			
5.36E-04	0.82	2.45E-02	0.64	902.44	0.00	0.00	undefined	1	LDGAKGDAGpAGPKGEPGSpGENGAPGQmGPRG	Collagen alpha-1(I) chain	273	305	P02452	COL1A1	6.92			

1.39E-03	0.79	3.30E-02	0.57	765.40	0.00	0.00	undefined	1	LDGAKGDAGpAGpKGEPGSpGENGAPGQmGPRG	Collagen alpha-1(I) chain	273	305	P02452	COL1A1	7.27			
1.39E-03	0.79	3.30E-02	0.57	231.30	0.00	0.00	undefined	1	GPQGFQGPPGEPGEPGASGPMGpRGpPGPPGKN	Collagen alpha-1(I) chain	197	229	P02452	COL1A1	2.64			
2.09E-04	0.89	1.71E-02	0.14	13.08	0.86	259.01	0.05	0	PGATGFPGAAGRVGpPGSNGNpGPpG	Collagen alpha-1(II) chain	893	918	P02458	COL2A1	2.93			
2.94E-04	0.90	2.10E-02	0.36	89.21	1.00	402.35	0.22	0	GpSGpAGARGIQGpQGPRGDKGEAGEPGER	Collagen alpha-1(II) chain	1098	1127	P02458	COL2A1	3.50			
1.17E-03	0.85	3.30E-02	0.29	223.18	0.93	789.59	0.28	0	GPAGApGpQGFQGNpGEPG	Collagen alpha-1(II) chain	213	231	P02458	COL2A1	3.38			
1.60E-03	0.85	3.66E-02	0.64	245.54	1.00	598.82	0.41	0	PVGpSGKDGANGIpG	Collagen alpha-1(II) chain	1168	1182	P02458	COL2A1	3.43			
1.41E-03	0.83	3.33E-02	0.79	100090.90	0.21	64.97	1540.57	1	GPpGADGQpGAKGEQGEAGQKGDAGApGPQGPSGAPGPQGP	Collagen alpha-1(II) chain	837	877	P02458	COL2A1	2.65			
1.95E-04	0.86	1.71E-02	0.71	2613.90	0.00	0.00	undefined	1	GEQGEAGQKGDAGAPGpQGpSGApGPQGp	Collagen alpha-1(II) chain	849	877	P02458	COL2A1	2.41			
1.95E-04	0.86	1.71E-02	0.00	0.00	0.71	89.47	0.00	0	PGpKGDAGAPG	Collagen alpha-1(III) chain	659	669	P02461	COL3A1	2.25			
4.31E-04	0.86	2.45E-02	0.14	4.56	0.79	66.60	0.07	0	DGKDGSpGEpG	Collagen alpha-1(III) chain	464	474	P02461	COL3A1	2.64			
2.25E-03	0.79	4.90E-02	0.07	50.10	0.64	681.96	0.07	0	NDGApGKNGERGGpGGPG	Collagen alpha-1(III) chain	586	603	P02461	COL3A1	3.01			
4.46E-04	0.87	2.45E-02	0.14	8.76	0.86	73.97	0.12	0	ApGKNGERGGpG	Collagen alpha-1(III) chain	589	600	P02461	COL3A1	2.63			
9.65E-04	0.86	3.30E-02	0.50	188.46	0.93	1248.65	0.15	0	ERGEAGIpGVpGAKGEDGKDGSPGEpGANG	Collagen alpha-1(III) chain	448	477	P02461	COL3A1	6.51			1
1.99E-04	0.90	1.71E-02	0.29	110.56	0.93	660.43	0.17	0	pGSQGESGRpGpPG	Collagen alpha-1(III) chain	554	567	P02461	COL3A1	2.36			
1.46E-03	0.84	3.41E-02	0.29	63.86	0.86	254.85	0.25	0	GEpGKNGAKGEpGP	Collagen alpha-1(III) chain	432	445	P02461	COL3A1	3.87			

1,14E-03	0,85	3,30E-02	0,21	83,60	0,93	318,97	0,26	0	ERGEAGIpGVpGAKGEDGKDGSpGEpG	Collagen alpha-1(III) chain	448	474	P02461	COL3A1	4,33			
1,45E-03	0,85	3,40E-02	0,43	122,16	1,00	246,50	0,50	0	SEGSPGHpGQPGpPGpPGApGP	Collagen alpha-1(III) chain	1174	1195	P02461	COL3A1	4,89			
1,22E-03	0,83	3,30E-02	0,71	328,25	0,14	3,29	99,77	1	GEPGGKGERGApGEKGEGGppGVAGpPGGS	Collagen alpha-1(III) chain	819	848	P02461	COL3A1	2,07			
1,95E-04	0,86	1,71E-02	0,71	3248,96	0,00	0,00	undefined	1	pGARGLpGPpG	Collagen alpha-1(III) chain	875	885	P02461	COL3A1	3,14			
1,39E-03	0,79	3,30E-02	0,57	9400,14	0,00	0,00	undefined	1	QpGEKGSPGAQGppGApG	Collagen alpha-1(III) chain	928	945	P02461	COL3A1	2,71			
1,39E-03	0,79	3,30E-02	0,57	982,45	0,00	0,00	undefined	1	GPSGSpGKDGppGpAGNTGAPGSP	Collagen alpha-1(III) chain	894	917	P02461	COL3A1	2,95			
1,39E-03	0,79	3,30E-02	0,57	2161,97	0,00	0,00	undefined	1	PpGSNGNpGPpGPSGSPGKDGPpGP	Collagen alpha-1(III) chain	883	907	P02461	COL3A1	3,31			
6,65E-05	0,89	1,43E-02	0,79	4651,40	0,00	0,00	undefined	1	GGAGPpGPEGGKGAAGpPGPpGAAGTpG	Collagen alpha-1(III) chain	696	723	P02461	COL3A1	3,88			
1,39E-03	0,79	3,30E-02	0,57	242,58	0,00	0,00	undefined	1	AGERGAPGFRGPAGPNGIPGEKGpAGERGA	Collagen alpha-1(III) chain	482	511	P02461	COL3A1	8,41			
5,36E-04	0,82	2,45E-02	0,64	1368,83	0,00	0,00	undefined	1	AGApGPAGSRGAPGpQGpRGDKGETGERGAAG	Collagen alpha-1(III) chain	1073	1104	P02461	COL3A1	3,07			
5,36E-04	0,82	2,45E-02	0,64	5796,32	0,00	0,00	undefined	1	SPGGKGDRGENGSPGAPGAPGHPGPpGPVGPAGKSGDRGESGPAGPAGA	Collagen alpha-1(III) chain	1027	1075	P02461	COL3A1	5,95			
1,95E-04	0,86	1,71E-02	0,71	2072,17	0,00	0,00	undefined	1	KGPPGPPGPAGEPGKPGAPGKPGTpG	Collagen alpha-1(IX) chain	298	323	P20849	COL9A1	2,35			
1,09E-04	0,90	1,71E-02	0,07	1,81	0,86	31,25	0,06	0	KGNSGGDGpAGPPGER	Collagen alpha-1(V) chain	933	948	P20908	COL5A1	2,09			
6,38E-04	0,84	2,73E-02	0,71	4068,77	0,07	3,93	1035,31	1	GPpGVTGMDGQpGpKGNVGPQGEPGpP	Collagen alpha-1(V) chain	694	720	P20908	COL5A1	3,50			
1,39E-03	0,79	3,30E-02	0,57	3827,72	0,00	0,00	undefined	1	LPGSpGpDGPpGPMGpP	Collagen alpha-1(V) chain	1451	1467	P20908	COL5A1	2,36			

1,39E-03	0,79	3,30E-02	0,57	3002,16	0,00	0,00	undefined	1	PGDKGDDGEPGQTGSPGPTGEpGpSGPp	Collagen alpha-1(V) chain	1362	1389	P20908	COL5A1	2,99			
5,36E-04	0,82	2,45E-02	0,64	3961,46	0,00	0,00	undefined	1	GPPGDKGDDGEPGQTGSPGPTGEPGPSGPP	Collagen alpha-1(V) chain	1360	1389	P20908	COL5A1	2,62			
5,36E-04	0,82	2,45E-02	0,64	2867,36	0,00	0,00	undefined	1	TGmDGQPGPKGNVGPQGEPGPPGQQGNpGAQG	Collagen alpha-1(V) chain	699	730	P20908	COL5A1	2,01			
8,37E-04	0,87	3,23E-02	0,64	168,36	1,00	704,91	0,24	0	FPGQTGPRGEMGQp	Collagen alpha-1(VII) chain	2405	2418	Q02388	COL7A1	2,31			1
1,39E-03	0,79	3,30E-02	0,00	0,00	0,57	93,66	0,00	0	GQQGMFGQKGDEGARGFPGPPGPIGLQGLpGppGEKGENGDVGPM	Collagen alpha-1(XI) chain	1174	1218	P12107	COL11A1	2,89			
7,10E-06	0,99	8,74E-03	0,29	22,61	1,00	461,68	0,05	0	EPGppGQQGNpGPQ	Collagen alpha-1(XI) chain	686	699	P12107	COL11A1	2,85			
1,00E-03	0,84	3,30E-02	0,79	3473,75	0,21	19,18	181,11	1	ppGDDGpKGNPGpVGFPGDPGPPGEPGP	Collagen alpha-1(XI) chain	1298	1325	P12107	COL11A1	2,88			
5,36E-04	0,82	2,45E-02	0,64	208,94	0,00	0,00	undefined	1	VGGDKGEDGDPGQpGPpGPSGEAGPpGP	Collagen alpha-1(XI) chain	1331	1358	P12107	COL11A1	3,23			
1,39E-03	0,79	3,30E-02	0,57	3262,47	0,00	0,00	undefined	1	GApGQPGmAGVDGPPGpKGNMGPQGEPGPPGQQGNPGPQGLPGPQGP	Collagen alpha-1(XI) chain	661	707	P12107	COL11A1	2,81			
5,36E-04	0,82	2,45E-02	0,64	597,92	0,00	0,00	undefined	1	GApGQpGMAGVDGpPGPKGNMGPQGEPGPPGQQGNPGPQGLPGPQGp	Collagen alpha-1(XI) chain	661	707	P12107	COL11A1	2,97			
6,34E-04	0,88	2,73E-02	0,43	316,36	1,00	1386,04	0,23	0	QPGDKGERGAAGEQGpDGpKGSKGEPGKGEM	Collagen alpha-1(XIII) chain	413	443	Q5TAT6	COL13A1	4,82			
1,39E-03	0,79	3,30E-02	0,57	1637,52	0,00	0,00	undefined	1	GDAGNSIGGGRGEPGPPGLPGPPGPKGEAGVDGQVGppG	Collagen alpha-1(XIII) chain	374	412	Q5TAT6	COL13A1	3,35			
6,65E-05	0,89	1,43E-02	0,79	9598,15	0,00	0,00	undefined	1	GPEGPSGKpGINGKDGIPGAQGImGKpGDRGpKGERGDQGIP	Collagen alpha-1(XIX) chain	917	958	Q14993	COL19A1	4,03			1
6,72E-04	0,88	2,85E-02	0,93	44249,55	0,93	2114,14	20,93	1	GpPGPpGPpGPpA	Collagen alpha-1(XV) chain	1096	1108	P39059	COL15A1	5,33			
5,87E-04	0,86	2,63E-02	0,79	3863,16	0,21	11,22	344,31	1	GKpGTDVFMGPPGSPGEDGPAGEPGPpG	Collagen alpha-1(XV) chain	634	661	P39059	COL15A1	2,69			

1,39E-03	0,79	3,30E-02	0,57	1807,36	0,00	0,00	undefined	1	KGEKGDRGDAGQKGERGEpGGGGFFGSSLpGpP	Collagen alpha-1(XVIII) chain	1324	1356	P39060	COL18A1	2,33			
5,07E-05	0,94	1,43E-02	0,21	42,96	1,00	339,39	0,13	0	GPPGPPGARGPPGDTGKDGP	Collagen alpha-1(XXIII) chain	187	206	Q86Y22	COL23A1	2,11			
5,02E-04	0,88	2,45E-02	0,36	138,83	0,93	657,87	0,21	0	KGDpGDpGpPGTHGNPGI	Collagen alpha-1(XXVIII) chain	244	261	Q2UY09	COL28A1	2,89			
1,30E-03	0,81	3,30E-02	0,64	1444,59	0,07	0,44	3283,16	1	PMGIpGIGSQGEQGIQGpIGpPGPQGPA	Collagen alpha-1(XXVIII) chain	445	472	Q2UY09	COL28A1	3,55			
4,33E-04	0,85	2,45E-02	0,07	1,01	0,71	69,37	0,01	0	DGppGRDGQpGHKGERGYpG	Collagen alpha-2(I) chain	933	952	P08123	COL1A2	1,94			
7,50E-04	0,85	3,06E-02	0,14	11,88	0,79	149,45	0,08	0	NGApGEAGRDGNpGNDGpPG	Collagen alpha-2(I) chain	918	937	P08123	COL1A2	4,46			
6,22E-04	0,87	2,73E-02	0,29	29,42	0,86	297,71	0,10	0	GpAGpRGERGPPGESG	Collagen alpha-2(I) chain	583	598	P08123	COL1A2	2,89			
7,05E-05	0,94	1,43E-02	1,00	2482,82	0,43	37,27	66,62	1	AVGPpGFAGEKGPSGEAGTAGPpGTpGPQG	Collagen alpha-2(I) chain	836	865	P08123	COL1A2	2,47			
1,62E-03	0,84	3,69E-02	0,79	4917,24	0,43	49,13	100,09	1	GISGPpGPpGPAGKEG	Collagen alpha-2(I) chain	802	817	P08123	COL1A2	4,73			
5,26E-04	0,84	2,45E-02	0,71	12659,78	0,07	2,95	4291,45	1	GpAGKDGRTGHPGTVGPAGIRGPQGHQGPAGPPGPPGPPGPPGVSGGGY	Collagen alpha-2(I) chain	1060	1108	P08123	COL1A2	2,77			
1,39E-03	0,79	3,30E-02	0,57	2211,45	0,00	0,00	undefined	1	GPpGPPGRDGEDGPTGPPGPPGpPGPpG	Collagen alpha-2(I) chain	45	72	P08123	COL1A2	2,44			
1,39E-03	0,79	3,30E-02	0,57	249,45	0,00	0,00	undefined	1	ISGPpGPpGPAGKEGLRGPRGDQGPVG	Collagen alpha-2(I) chain	803	829	P08123	COL1A2	3,15			
5,36E-04	0,82	2,45E-02	0,64	847,68	0,00	0,00	undefined	1	ARGPAGpPGKAGEDGHpGKPGRpGERG	Collagen alpha-2(I) chain	131	157	P08123	COL1A2	3,22			
1,95E-04	0,86	1,71E-02	0,71	1994,23	0,00	0,00	undefined	1	VNGApGEAGRDGNpGNDGpPGRDGQpGHK	Collagen alpha-2(I) chain	917	945	P08123	COL1A2	2,35			
1,39E-03	0,79	3,30E-02	0,57	1125,07	0,00	0,00	undefined	1	ARGEpGNIGFPGPKGpTGDpGKNGDKGHAG	Collagen alpha-2(I) chain	485	514	P08123	COL1A2	4,68			

1,39E-03	0,79	3,30E-02	0,57	744,62	0,00	0,00	undefined	1	TGAKGAAGLpGVAGApGLpGPRGIpGPVGAAGATGARG	Collagen alpha-2(I) chain	306	343	P08123	COL1A2	3,07			1
3,64E-04	0,86	2,45E-02	0,07	23,60	0,79	356,22	0,07	0	SGEPGpKGQQGVRGEpGYPGPSGDAGAPGVQGYPGPPG	Collagen alpha-2(IX) chain	459	496	Q14055	COL9A2	3,95			
1,52E-03	0,83	3,53E-02	0,14	44,13	0,79	299,04	0,15	0	PGpKGEpGKAGpDGpDG	Collagen alpha-2(IX) chain	62	78	Q14055	COL9A2	1,99			
7,20E-05	0,91	1,43E-02	0,86	6039,30	0,07	5,00	1207,86	1	VGMMGPPGpPGPPGYpGKQGpHGHpGPR	Collagen alpha-2(IX) chain	552	579	Q14055	COL9A2	1,96			
1,30E-03	0,81	3,30E-02	0,64	50838,53	0,07	4,19	12133,30	1	LGAVGmMGppGpPGPPG	Collagen alpha-2(IX) chain	549	565	Q14055	COL9A2	2,01			
1,33E-03	0,86	3,30E-02	0,86	5718,65	0,71	154,61	36,99	1	GPPGEPGPpGpPGPP	Collagen alpha-2(V) chain	1212	1226	P05997	COL5A2	2,89			
5,36E-04	0,82	2,45E-02	0,64	1844,60	0,00	0,00	undefined	1	PTGATGDKGPpGPVGpPGSNGpVGEPGPEGpAGN	Collagen alpha-2(V) chain	1021	1054	P05997	COL5A2	3,66			
5,36E-04	0,82	2,45E-02	0,64	8801,85	0,00	0,00	undefined	1	MGPLGPRGMPGERGRLGpQGApGQRGAHGMpGKPGPMG	Collagen alpha-2(V) chain	332	369	P05997	COL5A2	3,11			
4,41E-04	0,85	2,45E-02	0,07	7,53	0,79	106,48	0,07	0	KEGTkGDPGPpG	Collagen alpha-2(XI) chain	962	973	P13942	COL11A2	2,15			
1,67E-03	0,83	3,79E-02	0,79	1305,07	0,21	79,99	16,32	1	EPGPPGPSGLpGESYGSEKGApGDP	Collagen alpha-3(IV) chain	269	293	Q01955	COL4A3	3,43			
1,39E-03	0,79	3,30E-02	0,57	634,20	0,00	0,00	undefined	1	PGTpGNEGLDGpRGDPGQPGpP	Collagen alpha-3(IV) chain	779	800	Q01955	COL4A3	2,41			
5,36E-04	0,82	2,45E-02	0,64	3768,39	0,00	0,00	undefined	1	PGPAGEKGNKGSKGEPGpAGSDGLpGLKGKRGDSGSPA	Collagen alpha-3(IV) chain	1402	1439	Q01955	COL4A3	2,71			
8,08E-04	0,86	3,18E-02	0,21	66,72	0,93	324,57	0,21	0	mPGFKGpTGYKGEQGEVGKDGEKGDpGpPG	Collagen alpha-3(IX) chain	188	217	Q14050	COL9A3	2,76			
1,38E-04	0,92	1,71E-02	0,93	5266,62	0,50	56,43	93,33	1	GpAGPpGpPGPpG	Collagen alpha-3(IX) chain	557	569	Q14050	COL9A3	4,99			
1,02E-03	0,83	3,30E-02	0,71	51836,26	0,14	18,71	2770,51	1	pGPAGPpGpPGPpG	Collagen alpha-3(IX) chain	556	569	Q14050	COL9A3	4,83			

8,40E-04	0,85	3,23E-02	0,79	913,40	0,21	26,62	34,31	1	GPpGPpGFPGDPGPPG	Collagen alpha-3(V) chain	407	422	P25940	COL5A3	3,87			
1,39E-03	0,79	3,30E-02	0,57	202,48	0,00	0,00	undefined	1	GAQGpPGSAGPPGYPGPRGVKGTSGNRGLQGEKGEKGE	Collagen alpha-3(V) chain	695	732	P25940	COL5A3	2,56			
5,83E-05	0,91	1,43E-02	0,86	1003,29	0,07	7,24	138,58	1	GQDGIPGPAGQKGEpGQPGFGNpGppGL	Collagen alpha-5(IV) chain	1170	1197	P29400	COL4A5	2,25			
1,39E-03	0,79	3,30E-02	0,00	0,00	0,57	86,27	0,00	0	GDRGSGGAqGPAGApGLVGpLGp	Collagen alpha-6(IV) chain	516	538	Q14031	COL4A6	3,05			
7,06E-04	0,87	2,93E-02	0,36	102,92	1,00	305,26	0,34	0	SGpPGFPGLGTTGEKGE	Collagen alpha-6(IV) chain	273	289	Q14031	COL4A6	2,16			
1,17E-03	0,86	3,30E-02	0,93	4202,88	0,43	198,08	21,22	1	GPpGPPGpSSNQG	Collagen alpha-6(IV) chain	1277	1289	Q14031	COL4A6	3,46			
3,71E-05	0,93	1,43E-02	0,07	9,62	0,93	182,70	0,05	0	DELPAKDDPDAPLQPVTP	Complement C4-A	1423	1440	P0C0L4	C4A	2,93			
6,19E-05	0,94	1,43E-02	0,50	69,76	1,00	433,08	0,16	0	NRNPGSSGTGGTATWKPGSSGP	Fibrinogen alpha chain	307	328	P02671	FGA	3,53			
7,02E-04	0,87	2,93E-02	0,36	42,46	0,86	255,42	0,17	0	SEADHEGTHSTKRG	Fibrinogen alpha chain	609	622	P02671	FGA	4,30			
5,21E-04	0,87	2,45E-02	0,86	1033,68	0,29	29,87	34,61	1	PDWGTFEEVSGNVSPGTR	Fibrinogen alpha chain	408	425	P02671	FGA	6,48			
2,42E-04	0,88	1,83E-02	0,79	1370,89	0,14	4,55	301,29	1	PGSSGTGGTATWKPGSSGPGS	Fibrinogen alpha chain	310	330	P02671	FGA	2,37			
1,39E-03	0,79	3,30E-02	0,57	3114,28	0,00	0,00	undefined	1	TESRGSESGIFTNTKESSSHHPGIAEFPSRG	Fibrinogen alpha chain	544	574	P02671	FGA	6,92			
3,22E-04	0,89	2,23E-02	0,86	34261,24	0,43	138,08	248,13	1	DKKREEAPSLRPAPPPISGGGY	Fibrinogen beta chain	50	71	P02675	FGB	5,24			
1,39E-03	0,79	3,30E-02	0,57	2742,38	0,00	0,00	undefined	1	DKKREEAPSLRPAPPPISGGGYRARP	Fibrinogen beta chain	50	75	P02675	FGB	4,67			
1,10E-03	0,86	3,30E-02	0,57	1029,52	0,93	3432,54	0,30	0	WVGTGASEAEKTGAQEL	Gelsolin	605	621	P06396	GSN	4,55			1

7,72E-04	0,83	3,10E-02	0,71	7080,55	0,07	37,32	189,73	1	AAHLPAEFTPAVHASLDKFLASV	Hemoglobin subunit alpha	111	133	P69905	HBA1	2,36			
1,22E-03	0,83	3,30E-02	0,71	90778,02	0,14	21,21	4279,96	1	AAHLPAEFTPAVHASLDKF	Hemoglobin subunit alpha	111	129	P69905	HBA1	6,11		1	
5,36E-04	0,82	2,45E-02	0,64	17381,93	0,00	0,00	undefined	1	AAHLPAEFTPAVHASLDKFLAS	Hemoglobin subunit alpha	111	132	P69905	HBA1	5,65			
5,36E-04	0,82	2,45E-02	0,64	1084,19	0,00	0,00	undefined	1	HLPAEFTPAVHASLDKFLASVS	Hemoglobin subunit alpha	113	134	P69905	HBA1	3,59			
5,36E-04	0,82	2,45E-02	0,00	0,00	0,64	40,10	0,00	0	WGKVNVDEV	Hemoglobin subunit beta	16	24	P68871	HBB	2,35			
1,22E-03	0,83	3,30E-02	0,71	2033,79	0,14	12,95	157,05	1	AHHFGKEFTPPVQAA	Hemoglobin subunit beta	116	130	P68871	HBB	4,52			
1,39E-03	0,79	3,30E-02	0,57	14966,71	0,00	0,00	undefined	1	VHLTPEEKSAVT	Hemoglobin subunit beta	2	13	P68871	HBB	3,91		1	
1,39E-03	0,79	3,30E-02	0,57	498,08	0,00	0,00	undefined	1	PEPTKSAPAPKKG	Histone H2B type 1-D	2	14	P58876	H2BC5	3,64			
9,31E-04	0,83	3,30E-02	0,07	6,20	0,71	279,41	0,02	0	GSGWSSSNGPHGSVSGQSSGFGHKSGSGQSSGYS	Hornerin	681	714	Q86YZ3	HRNR	4,23			
1,39E-03	0,79	3,30E-02	0,00	0,00	0,57	40,57	0,00	0	DVVPIEPH	Interleukin-1 receptor antagonist protein	72	79	P18510	IL1RN	2,32			
1,99E-04	0,88	1,71E-02	0,07	28,87	0,86	297,28	0,10	0	SGDSDDDEPPPLPRL	Membrane-associated progesterone receptor component 1	54	68	O00264	PGRMC1	4,77	1		
1,95E-04	0,86	1,71E-02	0,71	4308,09	0,00	0,00	undefined	1	GVTGKSGLSAGVTGKTGLSAGVTGTTGPS	Mucin-19	5987	6015	Q7Z5P9	MUC19	4,13			
6,65E-05	0,89	1,43E-02	0,79	142687,41	0,00	0,00	undefined	1	GKVEADIPGHGQEVL	Myoglobin	16	30	P02144	MB	2,81			
1,95E-04	0,86	1,71E-02	0,71	1099,61	0,00	0,00	undefined	1	DGEWEESEVKLKKS	Neuroblast differentiation-associated protein AHNAK	5697	5710	Q09666	AHNAK	2,97			

1,39E-03	0,79	3,30E-02	0,57	1028,26	0,00	0,00	undefined	1	WLNPDPSQ	Osteopontin	43	50	P10451	SPP1	2,32			
1,25E-05	0,98	9,54E-03	0,36	101,30	1,00	2364,75	0,04	0	EEKAVADTRDQADGSRASVDSGSSEEQGGSSRALVSTLVPL	Polymeric immunoglobulin receptor	607	647	P01833	PIGR	9,18			
1,93E-04	0,91	1,71E-02	0,50	80,61	1,00	349,90	0,23	0	FAEEKAVADTRDQADGS	Polymeric immunoglobulin receptor	605	621	P01833	PIGR	2,35			
1,89E-03	0,84	4,20E-02	0,36	130,13	1,00	522,34	0,25	0	FAEEKAVADTRDQADGSRASVDSGSSEEQGGSSRALVSTLVPL	Polymeric immunoglobulin receptor	605	647	P01833	PIGR	6,18			
7,74E-04	0,87	3,10E-02	0,29	1256,59	1,00	2706,28	0,46	0	EEKAVADTRDQADGSRASVDSGSSEEQGGSSRALVSTLVPLG	Polymeric immunoglobulin receptor	607	648	P01833	PIGR	7,92	1		1
3,76E-04	0,89	2,45E-02	0,43	719,88	1,00	1371,65	0,52	0	FAEEKAVADTRDQADGSRASVDSGSSEEQGGSSRALVSTLVPLG	Polymeric immunoglobulin receptor	605	648	P01833	PIGR	7,74			
4,33E-04	0,85	2,45E-02	0,07	1,41	0,71	231,61	0,01	0	MKENFPNF	Protein S100-A7	36	43	P31151	S100A7	2,20			
1,95E-04	0,86	1,71E-02	0,00	0,00	0,71	142,01	0,00	0	LGHPDTLNQGEFKEL	Protein S100-A9	26	40	P06702	S100A9	3,93			
1,95E-04	0,86	1,71E-02	0,71	472,34	0,00	0,00	undefined	1	TWASHEKMHEGDEGPGHHHKPGLG	Protein S100-A9	87	110	P06702	S100A9	5,11			
2,68E-04	0,90	1,98E-02	0,29	976,29	1,00	811,46	1,20	1	TGRGAEDSLADQAAN	Serum amyloid A-2 protein	87	101	P0DJI9	SAA2	3,15			
1,39E-03	0,79	3,30E-02	0,57	367,16	0,00	0,00	undefined	1	AAPGQEPPEHMAELQRNEQEQPLGQWHLS	Sulfhydryl oxidase 1	615	643	O00391	QSOX1	2,84			
5,36E-04	0,82	2,45E-02	0,64	5195,67	0,00	0,00	undefined	1	KTETQEKNPLPSKETIEQEKQAGES	Thymosin beta-4	20	44	P62328	TMSB4X	3,06			
1,44E-04	0,90	1,71E-02	0,21	29,23	0,86	1384,12	0,02	0	DQSRVLNLGPITR	Uromodulin	594	606	P07911	UMOD	4,03			
1,75E-04	0,90	1,71E-02	0,21	87,29	0,86	1658,81	0,05	0	IDQSRVLNLGPITR	Uromodulin	593	606	P07911	UMOD	5,83			
3,85E-04	0,89	2,45E-02	0,29	1153,87	1,00	5207,01	0,22	0	SGSVIDQSRVLNLGPIT	Uromodulin	589	605	P07911	UMOD	5,61			

1,85E-04	0,91	1,71E-02	0,29	1168,71	1,00	4272,78	0,27	0	SGSVIDQSRVLNLGPI	Uromodulin	589	604	P07911	UMOD	6,10			

Supplementary figure 1: The alignment of COL1A1 fragments significantly associated with polytrauma to the COL1A1 sequence.

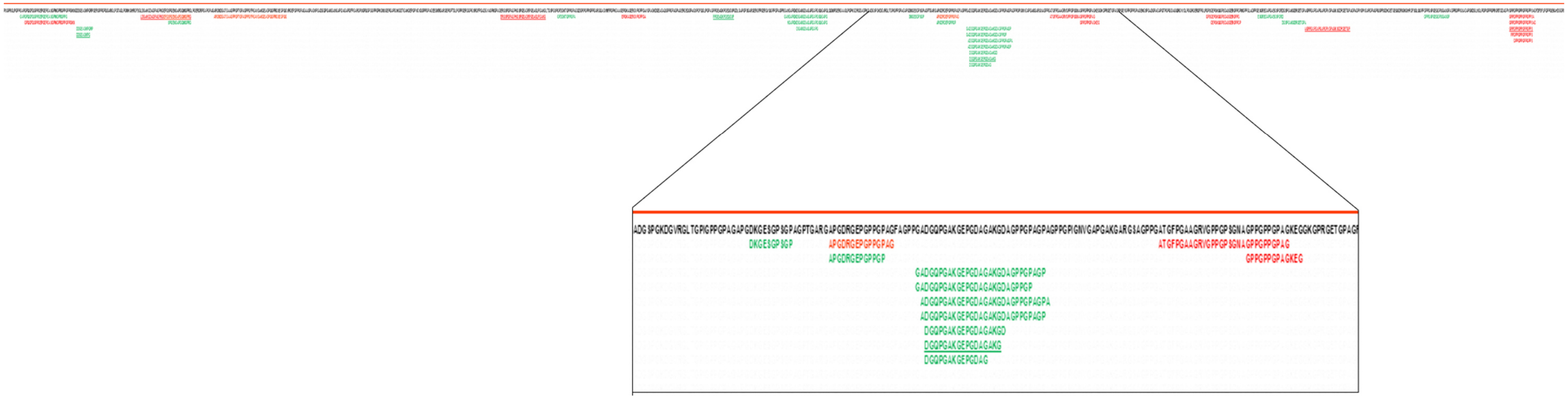

Supplement: Supplementary file 1 — Supplementary Material 1 [file 41598_2024_83878_MOESM1_ESM.pdf]
